# Assessing the detection and interaction of *Lawsonia intracellularis* and porcine circovirus 2 in low and high-performance wean-to-finish pig groups in different porcine reproductive and respiratory syndrome virus detection scenarios

**DOI:** 10.3389/fvets.2024.1535803

**Published:** 2025-01-15

**Authors:** Guilherme Cezar, Fernando L. Leite, Eduardo Fano, Reid Phillips, John Waddell, Kate Dion, Edison Magalhães, Giovani Trevisan, Gustavo Silva, Daniel C. Linhares

**Affiliations:** ^1^Department of Veterinary Diagnostic and Production Animal Medicine, Iowa State University, Ames, IA, United States; ^2^Boehringer Ingelheim Animal Health USA Inc., Duluth, GA, United States

**Keywords:** co-detection, oral fluid, PCV2, PRRSV, *Lawsonia intracellularis*, pathogen, mortality, average daily gain

## Abstract

**Introduction:**

Effective disease management strategies are essential for achieving optimal pig performance, ensuring high-quality animal health and welfare, and maintaining the economic viability of swine systems. Thus, understanding factors that lead to more or less severe disease are critically important. Porcine circovirus type 2 (PCV2) and *Lawsonia intracellularis* (*L. intracellularis*) are endemic pathogens in the U.S., affecting herds with varying degrees of subclinical and clinical disease and impact on performance. While these are common pathogens, their interaction with PRRSV and performance has seldom been investigated. This study investigated the detection dynamics of *L. intracellularis*, PRRSV, and PCV2, and their association with productivity impacts in wean-to-finish groups within a Midwest U.S. production system.

**Methodology:**

This observational field study involved batches of growing pigs from PRRSV-stable or PRRSV-negative sow farms. Oral fluids were collected longitudinally from weaning until market age, and tested using quantitative PCR for each of the aforementioned pathogens. The study included 36 batches with a total of 46,446 growing pigs, resulting in 4,000 oral fluid samples. Then, batches were categorized based on key performance indicators (mortality and average daily gain), PRRSV detection timing and total genomic copies of each pathogen.

**Results:**

Nineteen groups were characterized as high-performance and seventeen as low-performance. Mortality ranged from 5 to 9% in high-performance groups and 10.3–20.9% in low-performance groups. Average daily gain ranged from 0.68–0.86 kg in high-performance groups and 0.63–0.81 kg in low-performance groups. *L. intracellularis* and PCV2 were detected in most groups, with significant differences in detection rates between high and low-performance groups. Groups with relatively high genomic copies of PCV2 and *L. intracellularis* that had PRRSV detection presented higher mortality rates (15.75%).

**Discussion:**

This study expanded our understanding of PRRSV, PCV2, and *L. intracellularis* co-detections and their impact on swine populations.

## 1 Introduction

Effective disease prevention and control strategies are a major requirement to achieve optimum pig performance. Identifying and addressing the factors affecting pig performance is essential for ensuring animal health and welfare and maintaining the economic viability of swine enterprises. Adopting a holistic approach is crucial when investigating swine health issues to achieve optimum productivity (1, 2). Regarding controlling swine pathogens, this approach should consider all aspects of swine management, examining the interaction among them, housing, biosecurity, and health management practices (3).

The presence of multiple pathogens diagnosed in a pig flow compared to flows with few pathogens detected can cause higher mortality in wean-to-finish groups, mainly if the porcine reproductive and respiratory syndrome virus (PRRSV) is present among the flows (3). As described in the literature, the porcine respiratory disease complex (PRDC) is an example of an interaction among pathogens that can lead to severe respiratory lesions and cause economic losses in a farm (4). The etiology of PRDC varies between and within production systems and, over time, within the same system. On most farms with PRDC, one or two viruses, such as PRRSV or Influenza A virus, *Mycoplasma hyopneumoniae*, and several opportunistic bacteria (i.e., *Glaesserella parasui*, *Pasteurella multocida*, *Bordetella bronchiseptica*) work in combination to induce losses associated with respiratory disease (5). However, there is a lack of information about co-infections among pathogens that affect different systems (i.e., respiratory and enteric systems) and how they affect key performance indicators in swine farms.

PRRSV is known to cause systemic infection, affecting mainly the reproductive and respiratory systems. However, PRRSV infection can alter gut microbiome composition, an effect which has been related to strain virulence that becomes more pronounced during peaks of PRRS viremia (6). This microbiome disruption or dysbiosis could reduce colonization resistance to enteric pathogens as a reduction of beneficial anaerobic organisms has been observed along with increased *Proteobacteria* (6, 7). Changes to gut microbiome composition, in addition to playing a role in favoring enteric disease, have also been shown to be correlated to the porcine response to PRRSV and PCV2 (8, 9). Despite this, mixed infections involving respiratory and enteric pathogens affecting swine populations have been relatively understudied.

Porcine circovirus type 2 (PCV2) is another systemic virus that can cause different clinical manifestations, including PCV2-systemic disease, PCV2-reproductive disease, porcine dermatitis and nephropathy syndrome (PDNS), and PCV2-associated enteritis, depending on multiple factors (10, 11). Diarrhea, granulomatous enteritis, and lymphocyte depletion with granulomatous inflammation in Peyer’s patches are some of the findings associated with PCV2 enteric disease (10, 12). In a study conducted in Denmark, which examined tissues from 64 pigs with varying degrees of enteritis, it was found that 53.1% (34/64) of the pigs were infected with PCV2 and *Lawsonia intracellularis* (13), suggesting a possible association between these two pathogens. Proliferative enteropathy, also known as ileitis, is a clinical manifestation caused by *L. intracellularis*, an obligate intracellular organism (14, 15). This bacterium causes characteristic microscopic lesions, including the proliferation of intestinal epithelium and necrosuppurative enteritis (15, 16). Currently, the prevailing presentation of PCV2 or *L. intracellularis* infection is subclinical (10, 14, 17, 18). Even though no clear clinical signs are apparent for both subclinical presentations, field observations suggest that PCV2 and *L. intracellularis* vaccination enhance productive parameters (such as average daily gain, body condition, and carcass weight) even in scenarios of subclinical infections (10, 19, 20).

Even though there is evidence of PRRSV and PCV2 co-infections with multiple pathogens (21, 22), there are gaps in understanding how these viruses interact with enteric pathogens and affect production systems. These pathogens are known to be endemic in several U.S. swine farms, and there is a limited understanding of how the interaction among them might impact swine health. Moreover, there is a lack of information on the impact on productivity of the interaction among pathogens. The objectives of this study were to investigate the detection dynamics of *L. intracellularis*, PRRSV, and PCV2 and their association with the productivity impact in wean-to-finish groups from one production system located in the Midwest U.S.

## 2 Materials and methods

### 2.1 Study design

The observational field study involved seventy-five batches of growing pigs from PRRSV-stable with vaccination (*n* = 48) or PRRSV-negative (*n* = 27) from four sow farms classified as per the American Association of Swine Veterinarians (AASV) PRRSV herd classification (23) provided by a single production system. The study selected all batches of growing pigs based on specific eligibility criteria: (a) Pigs in each batch originated from either PRRSV positive-stable sow farms or PRRSV negative sow farms (farms consistently not producing viremic piglets at weaning age) (23); (b) All batches received vaccinations against *Lawsonia intracellularis* between 5–11 weeks of age and against PCV2 at the age of weaning (3 weeks) per system protocol from multiple manufacturers; (c) pigs were not vaccinated for PRRSV; (d) Pigs from sow farms with different PRRSV statuses were not mixed together; Finally, (e) the pigs were placed in the same region, which in this case was a high-density pig area in the state of Iowa, the U.S. state with the highest pig inventory, totaling 24 million among growing animals and sows (USDA, 2023).

**Figure 1:**
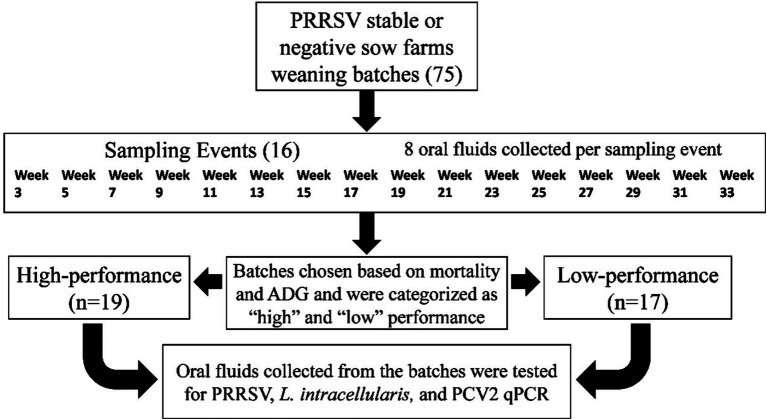
Summary of the study design.

Oral fluids were collected longitudinally (sampling the same batch of pigs consistently) from 2020 to 2021, from weaning age until they reached the market (Figure 1). The pens selected for oral fluids were spatially distributed among the barn, spacing the ropes evenly throughout the barn to access pens from the entrance until the barn end, adjusting for the different barn conformations, and collected every two weeks across 16 intervals from 3 to 33 weeks of age. At each sampling point, 8 oral fluids were collected. For each rope, 5 mL of oral fluids were squeezed using a resealable bag and transferred to 50 mL falcon tubes. Then, the samples were shipped to the Iowa State University Veterinary Diagnostic Laboratory for PCR testing, and aliquots were frozen and stored in −80 Celsius freezers.

Regarding group performance, each batch represents a cohort group in this longitudinal study, and the process involves combining data from both the periods before and after weaning into a single dataset, often called a master table, and was utilized for this analysis (2). This dataset contained a complete history of each production cycle, including details from before and after the animals were weaned, including mortality, average daily gain, sow farm source, and diagnostic history (2). In this study, “batches” refer to groups originating from breeding herds. After weaning, at about 21 days of age, they were moved to growing sites, where they remained for roughly six months. The management system follows an “all-in-all-out” approach, meaning a new group of pigs can only start once all the pigs from the previous group have been sold. The analysis in this study focuses on the mortality and average daily gain of each group during the entire growing period. The mortality was calculated by taking the difference between the number of pigs placed and the number of pigs remaining when marketed, divided by the number of pigs placed initially. The final average daily gain was calculated using the total weight gain during the growing period divided by the total number of days on feed.

After having the batches’ performance information, 36 out of the 75 groups were selected to test all of the oral fluids collected by PCR (Figure 1). These groups were selected based on mortality rates and average daily gain (ADG). The 75 groups were grouped in quantiles (1st through 4th quartiles) based on the mortality and ADG performance of the groups after they were marketed. R (R, v.4.2.3, R Core Team, Indianapolis, IN) quantile function using numeric vector probability 0 and 1 divided by 0.25 was utilized to create the quantiles. The extreme quantiles were chosen to classify the groups as Low-performance: high mortality (quantiles 3–4) and low ADG (quantiles 1–2), and the groups of High-performance had low mortality (quantiles 1–2) and high ADG (quantiles 3–4).In the group selection, two outliers belonging to the extreme quantiles were removed from the analysis after field veterinarians confirmed that these were groups with anomalies in the data, such as animals transferred to other facilities due to building issues. Mortality was prioritized over ADG if the groups had the same quantile classification. As an example, if the group “a” and “b” were classified as quantile 4 mortality and quantile 2 ADG, and the mortality of group “a” was equal to 12% and group “b” was 11%, the group “a” was included in the analysis. In case the mortality was similar, and the difference was only in decimals cases (i.e., 12.36 and 12.11%), a group was prioritized to be chosen if a tissue sample was submitted for diagnostic analysis to Iowa State University Veterinary Diagnostic Laboratory (ISU-VDL) for further evaluation by a diagnostician resulting in more diagnostic information for the group.

The Iowa State University Institutional Animal Care and Use Committee (IACUC) reviewed and approved the study protocol on June 03, 2019, under log number 3–18–870-S. The oral fluids were collected from the animals from May 2020 until November 2021.

### 2.2 qPCR testing and categorizing batches of growing pigs according to PCR results

Frozen oral fluids aliquots from the 36 selected groups were thawed and tested by quantitative PCR to detect PRRSV RNA, *L. intracellularis* and PCV2 DNA. Individual oral fluid samples were tested for the presence of PRRSV RNA after extraction of 100 μL using the MagMAX Pathogen RNA/DNA kit (ThermoFisher Scientific, Waltham, MA, USA) and a Kingfisher Apex purification system (ThermoFisher Scientific) per the manufacturer’s instructions followed reverse transcription-quantitative polymerase chain reaction (RT-qPCR) for PRRSV or quantitative PCR (qPCR) for *L. intracellularis* and PCV2. The PRRSV RT-qPCR was conducted using the commercial VetMAX™ PRRSV NA&EU 3.0 kit (ThermoFisher Scientific) per manufacturer’s instructions. A sample was considered positive for PRRSV RNA if the cycle threshold (Ct) value was <37. The sample extracts were also tested at the ISU-VDL using a template extracted with the same method for the PRRSV RT-qPCR. The *L. intracellularis* and PCV2 qPCR conducted were ISU VDL-developed assays based on standard operating procedures. The samples were considered positive if *L. intracellularis* Ct values were < 35 and PCV2 Ct values were < 37. Genomic copies per mL of oral fluid samples were calculated using PRRSV RT-qPCR Ct values with a standard curve template provided by the kit and for *L. intracellularis* and PCV2 qPCR Ct values with standard curves developed at the ISU-VDL using synthesized and quantified template sequence.

Batches of growing pigs were classified based on the timing of the first PRRSV detection, which were categorized as nursery (3–9 weeks of age), early finish (11–13 weeks of age), and late finish (14 weeks onward). This classification based on the first detection created a variable “PRRSV first detection” as a categorical variable for statistical modeling. In addition, based on the qPCR results of *L. intracellularis,* PCV2, and PRRSV, three additional categories were created. The categories were named “total *Lawsonia genomic copies,” “total PCV2 genomic copies,” and “total PRRSV genomic copies.”* These categories were the sum of all the qPCR genomic copies detected for each pathogen throughout the whole period the animals where the oral fluids were collected and tested (i.e., if at each of the 16 sampling points, a total of 1,000,000 PCV2 genomic copies were detected in all oral fluid samples; this group had a total of 16 million genomic copies). Then, the groups were divided into quantiles based on the log 10 transformation of the total genomic copies of the pig groups, with quantile 1 representing the groups with lower amounts of genomic copies and quantile 4 representing the groups with higher amounts in this dataset.

### 2.3 Statistical analysis

The unit for the statistical analysis was the pig batch, which comprised 36 groups. Regression models (R, v.4.2.3, R Core Team) were used to evaluate the differences in oral fluid PCR positivity for *Lawsonia* and PCV2 at each sampling age, cumulative mortality, and ADG among the High/Low performance and PRRSV detection groups.

Binomial regression models were constructed to analyze each sampling event’s proportion of oral fluid-positive samples of the performance groups for each pathogen. This proportion was computed by dividing the number of PCR-positive oral fluid samples by the total number of oral fluids tested (weight) in each performance group’s sampling point (subset). Binomial modeling was used, adding the variable “PRRSV first detection.” *p*-values and positive rates with 95% confidence intervals (CI) were reported. The average percentage of positive oral fluid samples within a week age in the two performance groups was assessed and plotted over time to visualize the detection dynamics in the two performance groups.

Generalized linear mixed regression models were used to compare key closeout productivity indicators between high and low-performance groups, and PRRSV first detection groups. For mortality, binomial regression models were used to analyze the mortality rate between study groups (performance groups and PRRSV first detection groups), considering the groups with at least a *L. intracellularis*, PRRSV, and PCV2 detection in oral fluid at any sampling point. This was done by considering the counts of dead pigs, adjusted by the number of pigs placed, as a weight variable. Additionally, linear mixed regression models were used to analyze differences in average daily gain as a response variable among the same groups. Both models included the performance category and PRRSV first detection as fixed effects with interaction, while sow farm sources were considered as random effects. Group outcomes (ADG and mortality) were compared through pairwise comparisons using the Tukey–Kramer test. Additionally, separate models for ADG and mortality following the same methodology using only the *L. intracellularis* positive groups and the PCV2 positive groups, evaluating their performance under different PRRSV first-entry scenarios.

To assess the pathogens’ interaction and impact on the groups’ mortality, binomial regression models using the mortality outcome were utilized, and the variables *total PCV2 genomic copies, total Lawsonia intracellular genomic copies, and total PRRSV genomic copies*, already in the quantile format, were added separately, assessing the interaction of each of them separately in each model (i.e., total Lawsonia * total PCV2; total PCV2 * total PRRSV; total Lawsonia*total PRRSV) as categorical variables. In the binomial model, the effect of each quantile combination was evaluated through pairwise comparisons using the Tukey–Kramer test. This involved comparing the probability of mortality proportion and average daily gain performance between different quantile combinations assessed as explanatory variables. *p*-values, mortality proportion, ADG least-square means, and their corresponding 95% confidence intervals were reported for all the analyses. Quantiles of deviance residuesand binned residual plots were utilized to assess the model’s overall fit.95% of the predicted probability plotted against the residuals had to be within the confidence limits to indicate model fit (24). Regarding deviance residue, the general linear deviance range was 3 and − 3 to consider a model fit (25). The outcomes of the statistical analysis were then connected to Microsoft Power BI (Power Business Intelligence; Microsoft, Redmond, WA), enabling a user-friendly data visualization.

## 3 Results

The study included 36 batches with a total of 46,446 growing pigs that were followed up over time, collecting 4,000 oral fluid samples tested for PRRSV, *Lawsonia intracellularis*, and PCV2 by RT-qPCR or qPCR. Groups of growing pigs within the same production system came from four different sow farms with two different PRRSV statuses (stable and negative).Among these 36 groups, 29 received *L. intracellularis* oral vaccines and 7 received injectable vaccines.

### 3.1 Groups definition

Based on the performance of (mortality and ADG) 19 groups were characterized as High and 17 as Low-performance groups.The mortality range within the performance groups was 5–9% (High-performance) and 10.3–20.9% (Low-performance). The average daily gain was 0.68–0.86 kg (High) and 0.63–0.81 kg (Low). The binomial model with mortality as an explanatory variable indicated a mean probability of mortality of 13.94% (CI 95%: 13.07, 14.87) in the low-performance group, statistically higher (*p*-value <0.001) compared with 7.01% (CI 95%: 6.49, 7.56) mortality in the high-performance group. The linear mixed model with ADG as an explanatory variable indicated a mean of 0.73 kg (CI 95%: 0.69, 0.77) daily gain in the high-performance group, statistically higher (p-value = 0.0261) than the low-performance group 0.71 kg (CI 95%: 0.67, 0.75).

Regarding the PRRSV first detection, 35 of the 36 groups had lateral introduction of PRRSV. Nine groups had the first PRRSV detection in oral fluids during 3–9 weeks of age (nursery), followed by 14 groups in the weeks of age 11–13 (early finish) and 12 groups with detection at 15–33 weeks of age (late finish), and only one group without PRRSV detection via oral fluid. The groups with PRRSV detection in the early and late finish phases had higher mortality than those without PRRSV detection and first detection at the nursery phase. However, there was no statistical difference in ADG among the groups (*p*-value = 0.5867). Of the 36 groups, 31 were positive for PCV2 and *L. intracellularis* at some sampling point. Two groups were positive for PCV2 and negative for *L. intracellularis* and 1 group was positive for *L. intracellularis* and negative for PCV2. Only two groups were negative for PCV2 and *L. intracellularis* in all the sampling points. Also, 15 groups (4 High-performance and 11 Low-performance) had tissue submissions at the Iowa State University Veterinary Diagnostic Laboratory for further clinical evaluation by diagnosticians. However, none of these groups had confirmed diagnostic codes (26) for *L. intracellularis* or PCV2. The performance of the groups where *L. intracellularis* and/or PCV2 were detected under different PRRSV detection scenarios is described in Table 1.

**Table 1:** Mortality and average daily gain differences for groups where *Lawsonia intracellularis* and PCV2 were detected in at least one sampling point under different PRRSV first detection scenarios.

PRRSV first detection (Performance groups)	Mortality (%) (95% CI)		ADG (kg) (95% CI)	
	High	Low	High	Low
Nursery (High = 7; Low = 2)	7.69^b^ (6.65, 8.87)	13.89^c^ (11.44, 16.78)	0.71^ab^ (0.67, 0.76)	0.77^ab^ (0.63, 0.90)
Early finish (High = 5; Low = 8)	6.55^ab^ (5.57, 7.70)	14.94^c^ (13.31, 16.74)	0.76^b^ (0.72, 0.80)	0.69^a^ (0.64, 0.73)
Late finish (High = 3; Low = 5)	6.03^a^ (4.94, 7.34)	13.67^c^ (12.04, 15.49)	0.75^ab^ (0.68, 0.81)	0.69^ab^ (0.64, 0.74)

Different letters indicate a significant statistical difference (*p*-value < 0.05), separately for mortality and average daily gain, between groups where PCV2 and Lawsonia intracellularis were detected within the PRRSV first detection categories through pairwise comparison (Tukey–Kramer).

There were statistical differences (*p*-value <0.05) in mortality among all the combinations of PRRSV first entry and performance groups where *L. intracellularis* and PCV2 were detected. All the Low-performance groups had higher mortality than the High-performance. However, within the Low-performance groups, the PRRSV first-entry category had no statistical difference in mortality. In contrast, in the High-performance groups, the groups where PRRSV was first detected in the nursery phase (7.69%) had higher mortality than those where the virus was detected only in the late finish phase (6.03%). For ADG, there were no statistical differences among the High and Low-performance groups, except for when PRRSV was first detected in the early finish phase. The Higher-performance groups had higher ADG compared with Low-performance (*p*-value = 0.0352) (Table 1). The highest numeric mortality was in the group positive for *L. intracellularis* and PCV2, where PRRSV first detection occurred in the early finish phase with 20.89% mortality. The lowest mortality was present in the high-performance group positive for *L. intracellularis* and PCV2, with the PRRSV first detection occurring in the early finish phase, with 4.96% mortality. Also, for average daily gain, the lowest numerical value (0.66 kg) was in the Low-performance group positive for PCV2 and *L. intracellularis* with the first PRRSV detection in the late finish phase. The high-performance group with first PRRSV detection at the early finish phase and positives for *L. intracellularis* and PCV2 had the highest numerical ADG (0.82 kg).

### 3.2 Pathogen nucleic acid detection in oral fluids over time

**Figure 2:**
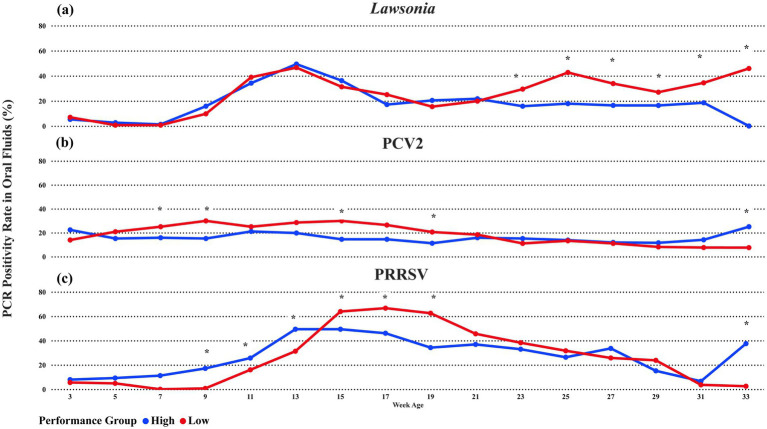
The average percentage of PCR-positive oral fluids for **(A)** *Lawsonia intracellularis*, **(B)** Porcine Circovirus 2, or **(C)** PRRSV between performance groups. The blue line represents High-performance groups, and the red line represents Low-performance groups. * oral fluid detection with a statistical difference at a significance level of 0.05 between the performance groups.

*L. intracellularis* DNA was detected in at least one sampling point in 31 of the 36 groups tested in this study. Both high and Low-performance groups had a peak in the oral fluid detection rate at week 13 of age (91 average days of age), with 45.5% (CI 95%: 37.7, 53.4) detection in the High-performance group and 50.7% (CI 95%: 42.6, 58.8) in the Low-performance groups. After the peak in the detection, both performance groups had a decrease in the positivity rate of oral fluids (Figure 2A). However, at week 23 (161 average days of age), the low-performance groups showed a statistically different oral fluid detection rate (*p*-value <0.05). The highest difference between the performance groups was identified in the week of age 33, where the lowest performance group had 46.4% of oral fluids positive for *L. intracellularis,* and the high-performance group had 0%. It is essential to highlight that all 31 positive groups had oral fluid samples collected until the week of age 27. Still, due to the pig marketing strategies of the production system, only 7 (two high and five low-performance groups) of these 31 groups had animals in the barns until the week of age 33.

PCV2 DNA was detected in at least one sampling point in 32 of the 36 groups tested in this study. Low-performance groups had an oral fluid detection rate from week 5 of age (35 average days of age) until week 21 of age (Figure 2B). The difference between the low and high-performance groups during this period was statistically significant at a 0.05 significance level at weeks 7, 9, 15, and 19 of age (Figure 2B). The highest detection difference occurred in weeks 9 and 15 of age, where the low-performance group had a 29.9% (CI 95%: 23, 37.8) oral fluid positivity rate compared with 15.1 (10.3; 21.7) and 14.5% (9.7; 21) in the high-performance groups, respectively. In addition, the high-performance groups had higher detection at 33 weeks of age. Again, only 7 (2 High and 5 Low performance) out of 32 were sampled at age 33 due to the pig marketing strategies. Still, the high-performance group had an oral fluid detection of 25% (CI 95%: 14.3, 66.1) compared with 6.6% (CI 95%: 1.6, 23.1) in the low-performance group. Also, numerically, the oral fluid detection rate of the high-performance was higher in the three weeks of age (first sampling point) and during weeks 23 until 33 (Figure 2B).

PRRSV RNA was detected in at least one sampling point in 35 of the 36 groups tested in this study. High-performance groups had an oral fluid detection rate from the beginning of sampling (week age 3) until week 13 of age, with statistical differences at weeks 9 (17.10%), 11 (25.66%), and 13 (49.34%) (Figure 2C). Then, from weeks 15 until 21, the Low-performance group had a statistical difference in oral fluid positivity compared with the High-performance achieving the highest positivity rate on week 17 with 66.67% (CI 95%: 61.4, 76.7). Interestingly, after weeks 15 until 21, with increased positivity in PRRSV, a sequence of weeks with increased positivity of *L. intracellularis* occurred from weeks 23 until 33 (Figures 2A, C). For PCV2, the High-performance groups had higher PRRSV detection at 33 weeks of age with the same number of High and Low-performance groups.

**Figure 3:**
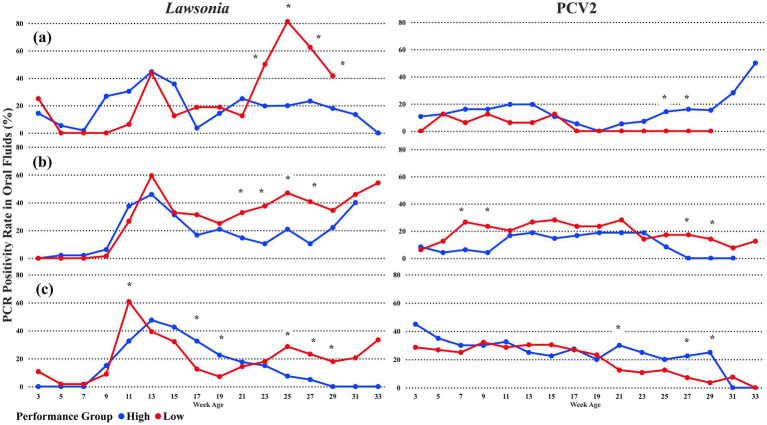
The average percentage of PCR-positive oral fluids for *Lawsonia intracellularis* and Porcine Circovirus type 2 among PRRSV detection groups. The blue line represents High-performance groups, and the red line represents Low-performance groups. **(A)** PRRSV 1st detection in nursery pigs; **(B)** PRRSV 1st detection in early finish pigs; and **(C)** PRRSV 1st detection in late finish pigs. * oral fluid detection with a statistical difference at a significance level of 0.05 between the performance groups.

Breaking down the pathogen detection by the first PRRSV introduction, *L. intracellularis* followed the same overall pattern of detection described previously. From week 23 until week 33, the low-performance groups predominantly had higher detection of *L.intracellularis* than the high-performance group, regardless of the time the first PRRSV detection occurred (Figures 3A–C). However, for the sampling points with statistical differences, the groups where PRRSV was first detected at the early finish phase had statistical differences at week age 21, with 32.8% (CI 95%: 22.47, 43.5) of oral fluid positive rate (Figure 3B). The statistical difference occurred at one sampling point before the overall detection (Figure 2) without breaking down the PRRSV detection categories. Also, when PRRSV was first detected at the late finish phase (Figure 3C), the high-performance group had a statistically higher oral fluid detection rate at the week ages 17 (32.5%) and 19 (22.5%) compared with the low-performance group. Moreover, the peak at the week of age 11 in all the groups was statistically higher in the Low-performance group, with 60.7% (CI 95%: 47.5, 72.5) oral fluid positive rate (Figure 3C).

For PCV2, the detection pattern was different among the PRRSV first detection scenarios. The early finish group (Figure 3B) follows a similar pattern identified in the overall PCV2 detection chart (Figure 2B) in the nursery phase with statistical differences at the week of ages 7 and 9. However, the low-performance groups had higher detection in the weeks of ages 27 and 29, mainly because of the 0% of positive oral fluids samples among the high-performance groups. On the other hand, when PRRSV was first detected in the nursery or late finish phase, a higher oral fluid positive rate for PCV2 was observed in the high-performance groups (Figures 3A,C). Differently than the overall PCV2 oral fluid detection (Figure 1B), the nursery and late finish groups demonstrated a higher detection rate of PCV2 in the high-performance groups compared with the low-performance groups, with statistical differences at sampling ages 21, 25, 27, and 29 (Figures 3A,C). From the sampling age 17 onward, there was no PCV2 detection in oral fluids among the low-performance groups when PRRSV first detection occurred in the nursery (Figure 3A).

### 3.3 Detection interaction among pathogens

For the RT-qPCR and qPCR results, *Lawsonia intracellularis* had a higher number of average total genomic copies throughout the whole production cycle in log_1o_ (6.55), followed by PRRSV (6.37) and PCV2 (6.19). Since the three pathogens had groups with no detection of PCV2, *L.intracellularis*, or PRRSV, the minimum number of genomic copies was 0 for all the pathogens. For *L.intracellularis*, three groups did not have detection; for PCV2, two groups and PRRSV, one group did not have detection on qPCR results in oral fluids. Regarding the maximum total number of genomic copies for each pathogen, *L.intracellularis* had the group with the entire highest number of genomic copies, with 7.80 genomic copies throughout the whole production cycle, followed by PCV2 with 7.50 genomic copies, and PRRSV with 7.04. Table 2 summarizes the quantile division of the 3 pathogens analyzed in this research based on the log 10 transformation of the total genomic copies.

**Table 2:** Distributions of the quantiles based on the total number of genomic copies throughout the whole production cycle of the group for *Lawsonia intracellularis*, PCV2, and PRRSV.

Pathogen	Quantile	Number of groups	Genomic copies (log 10)
*Lawsonia intracellularis*	1	9	0–5.11
*Lawsonia intracellularis*	2	9	5.12–5.67
*Lawsonia intracellularis*	3	9	5.68–6.13
*Lawsonia intracellularis*	4	9	6.14–7.81
PCV2	1	9	0–3.95
PCV2	2	9	3.96–4.69
PCV2	3	9	4.70–5.69
PCV2	4	9	5.70–7.51
PRRSV	1	9	0–5.93
PRRSV	2	9	5.94–6.14
PRRSV	3	9	6.15–6.44
PRRSV	4	9	6.45–7.04

**Figure 4:**
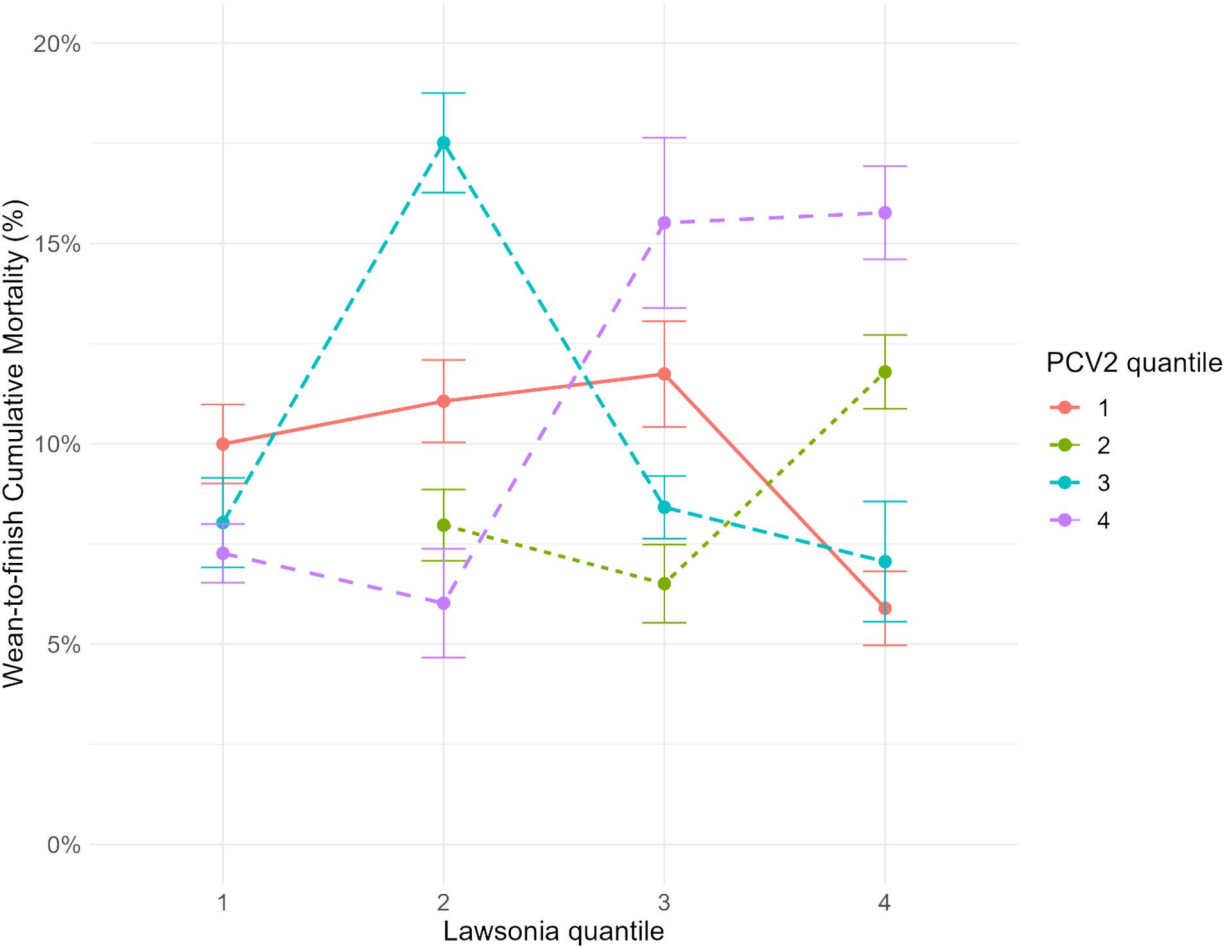
Interaction between *Lawsonia intracellularis* groups quantiles and Porcine circovirus type 2 groups quantiles at 95% confidence interval. Groups from quantile 1 had fewer total genomic copies, and quantile 4 had the highest amount of genomic copies throughout their production life.

Verifying the interactions among the PRRSV, *L. intracellularis*, and PCV2 and their association with mortality and ADG, the six models created for each pathogen-specific interaction were tested separately in the final model for mortality and ADG (PRRSV x PCV2; PCV2 x *L. intracellularis*; *L. intracellularis* x PRRSV). Regarding the mortality outcome, only the interaction between PCV2 and *L. intracellularis* was significant (*p*-value = 0.006). For ADG, none of the interactions among the pathogens were significant. In the pairwise comparison analysis for the interaction of PCV2 and *L. intracellularis* among the groups, the groups from the higher quantiles of *L. intracellularis* and PCV2 had statistically higher mortality than the other groups (Figure 4). For instance, three groups classified as quantile 4 of both PCV2 and *L. intracellularis* had the least square means mortality of 15.76% (CI 95%: 14.60, 16.92), followed by one group that belonged to quantile 3 of *L. intracellularis* and quantile 4 of PCV2 with 15.51% (CI 95%: 13.39, 17.64). Besides these two combinations, two groups of quantile 2 of *L. intracellularis* and quantile 3 of PCV2 had higher mortality with 17.51% (CI 95%: 16.27, 18.75). These groups had statistically higher mortality than the other quantile combinations in the pairwise comparison (Figure 4).

The groups from quantile 4 of *L. intracellularis* and quantile 1 of PCV2 had the lowest mortality, 5.89% (CI 95%: 4.96, 6.91). However, there was no statistical difference compared to the other six quantile combinations described in Table 3. In addition, there was no group with the combination of *L. intracellularis* quantile 1 and PCV2 quantile 2. It is essential to highlight that all these groups, analyzed by generalized mixed regression analysis and visualized by interaction plots, also had PRRSV detection throughout their life period except for one group from *L. intracellularis* quantile 2 and PCV2 quantile 1. In addition, from the 9 groups classified as quantiles 4 for PRRSV, only 2 groups were not classified as *L. intracellularis* or PCV2 quantiles 3 or 4, which means at least one of these pathogens had a high amount of total genomic copies when PRRSV had a higher number of genomic copies throughout the whole animal life cycle. However, an interaction among the three pathogens (PRRSV*Lawsonia*PCV2) could not be evaluated because of the limited sample size, and thus, the model would not converge due to the inexistence of quantile combinations.

**Table 3:** Distributions of the *Lawsonia intracellularis* and PCV2 quantiles combinations based on the total number of genomic copies throughout the whole production cycle of each group and its impact in mortality.

*Lawsonia* quantile	PCV2 quantile	Number of groups	Mortality (%) (95% CI)
1	1	3	9.99% (9, 10.98)^cd^
1	2	0	NA
1	3	2	8.03% (6.91, 9.14)^abc^
1	4	4	7.26% (6.53, 7.99)^ab^
2	1	3	11.06% (10.03, 12.09)^d^
2	2	3	7.96% (7.07, 8.86)^abc^
2	3	2	17.51% (16.27, 18.75)^f^
2	4	1	6.02% (4.66, 7.38)^ab^
3	1	2	11.74% (10.42, 13.05)^de^
3	2	2	6.5% (5.53, 7.48)^ab^
3	3	4	8.41% (7.63, 9.19)^bc^
3	4	1	15.51% (13.39, 17.64)^ef^
4	1	1	5.89% (4.96, 6.81)^a^
4	2	4	11.79% (10.87, 12.71)^de^
4	3	1	7.05% (5.55, 8.56)^abc^
4	4	3	15.76% (14.60, 16.92)^f^

^abcdef^ Different letters indicate a significant statistical difference (*p*-value < 0.05) in mortality between *Lawsonia intracellularis* and PCV2 quantiles combination groups through pairwise comparison (Tukey–Kramer). NA, without estimate.

## 4 Discussion

In this study, groups of pigs, which suffered a PRRSV introduction, with lower average daily gain (ADG) and higher mortality had higher levels of *L. intracellularis* detection. For PCV2, the higher detection followed a different pattern with groups with higher detection of PCV2 in both high- and low-performance groups. Binomial regression utilized in this study allows for modeling binary outcomes, such as the presence or absence of *L. intracellularis* and PCV2 detection in oral fluid samples (yes/no), while accounting for potential confounding variables (27). By employing this statistical approach, the relationship between predictor variables and the likelihood of *L. intracellularis* and PCV2 detection in oral fluid samples can be accessed, providing valuable insights into factors influencing these pathogens’ detection dynamics in oral fluid.

Among the 36 groups studied, 35 became PRRSV-positive at different times. When PRRSV was detected early in the finishing phase, there was an increase in the detection of both *L. intracellularis* and PCV2 in the low-performance groups, suggesting a possible association between PRRSV, *L. intracellularis,* and PCV2 in this specific phase. PRRSV and PCV2 target the host’s immune cells, disrupting their immune function. This disruption increases susceptibility to primary and secondary pathogens, significantly affecting host growth performance and increasing the incidence and lethality of associated diseases (28, 29). Also, PRRSV causes significant tissue lesions and inflammation, which might favor infections of other pathogens (21).

PRRSV infection does not significantly impair lymphocyte differentiation/maturation or cause severe lymphocyte failure or ablation, indicating that the host’s adaptive immune response is not compromised (28, 30). However, through various methods, PRRSV infection can impair healthy thymic function and affect immunological responses, lowering or modifying T cell development and delaying and weakening adaptive immune responses (28, 30). These immune disruptions caused by the virus can impact even vaccinated animals for other pathogens, such as Influenza A virus (31). Also, this viral-mediated suppression is known to disrupt the development of adaptive immunity, particularly in young pigs (29, 30), resulting in a low-performance impact on younger pigs (32). However, this study identified the groups with higher mortality when PRRSV was first detected in pigs older than 14 weeks, which might be associated with the other factors in the pig flow. In addition, there is evidence of *L. intracellularis* higher detection in oral fluids in the late finish phase (33), with higher circulation of PCV2 being a potential risk factor for *L. intracellularis* increased detection in the farms (33). This study also demonstrated higher detection of *L. intracellularis* occurring from the week of age 23 until the week of age 33, while either PRRSV, PCV2, or PCV2 and PRRSV were detected in the batches.

Interestingly, high-performance groups where PRRSV was first detected in the late finishing and nursery phase had higher PCV2 oral fluid detection from 21 weeks of age onward compared to low-performance groups. This might indicate that increased PCV2 detection in oral fluid without the manifestation of porcine circovirus-associated disease (PCVAD) does not necessarily affect performance in vaccinated pigs (34–36) under the conditions of the timing of PRRSV introduction. Also, PCV2 can be detected during extended periods in oral fluid, up to 98 days post-inoculation, without necessarily expressing clinical disease (37).

The interaction between PRRSV and PCV2 has been previously described in inoculation trials expressing disease in non-vaccinated animals (38, 39). Still, this study is limited in analyzing the oral fluid results and tissue submissions of groups selected by the practitioners for further investigation in diagnostic laboratories for microscopical lesions. Different co-infections and detection dynamics on the performance of pigs affected by multiple physiological systems might have occurred since it was a prospective epidemiological field study unable to control all pathogens affecting the animals. However, none of the 15 groups analyzed by the diagnosticians had evidence of PCV2-associated lesions.

In addition, in this study, a genomic copy quantification of each pathogen was performed for all groups of pigs throughout their time in the barn. Positive quantitative PCR results were summed for each pathogen in each group. Based on the total amount of genomic copies, the groups were classified into four quantiles to assess the interaction among PRRSV, *L. intracellularis*, and PCV2 quantification results in oral fluids. From the authors’ perspectives, this analysis in field conditions had not been performed before. Based on this analysis, groups with higher levels of *L. intracellularis* and PCV2 genomic copies statistically exhibited higher mortality rates (15.7%) than other groups (Figure 2). The association of *L. intracellularis* and PCV2 causing the clinical disease was described in Iberian pigs developing granulomatous enteritis and lymphadenitis under experimental conditions (12). Also, a study conducted in Italy observed the presence of co-detection between *L. intracellularis* and PCV2 cases through immunohistochemistry (40). In these *L. intracellularis* and PCV2 cases in Italy, the authors suggested that both hypotheses should be considered for these severe lesions associated with both pathogens: PCV2 as the primary agent causing severe immune system dysfunction, which worsens the pathogenicity of other agents or alternatively, the ability of *L. intracellularis* to prime PCV2 replication (40).

However, to confirm a possible relationship between *L. intracellularis* and PCV2, conventional pigs were inoculated with PCV2, *L. intracellularis*, or both under experimental conditions (41). Regardless of co-infection status, individual pigs inoculated with PCV2 developed severe PCVAD, characterized clinically by weight loss and microscopically by PCV2-antigen-associated severe lymphoid depletion and histiocytic replacement of most lymphoid tissues (41). This indicates that *L. intracellularis* might not enhance PCVAD in controlled conditions (41), but its co-detection in field conditions might have a different interaction. In addition, in this prospective field trial, the fact that PRRSV was also involved in this co-detection dynamics might play a role in the performance of the animals. The virulence of the PRRSV strain can influence microbiome alterations, which become more pronounced at peaks of PRRS viremia (6). The microbiome disruption and the intestinal barrier damage potentially favor the colonization of enteric pathogens (6, 9, 42).

Numerically, the group classified as *L. intracellularis* category two and PCV2 category 3 had the highest mortality. However, this classification applied to two groups also affected by the PRRSV lineage LC.5 (variant) 1–4-4 by ORF5 sequencing. This specific variant is known for its severe clinical outcomes in the field, causing higher losses in swine production systems (43–45). The study could not analyze the possibility of PRRSV diversity influencing these co-detection dynamics. Not all the flows had PRRSV ORF5 sequence data was not available for all of the groups in the study, as the only available sequences were from groups where the practitioners requested the sequencing test to be performed on groups with low Ct values. However, PRRSV diversity is an issue in the U.S. (46, 47), and as the L1C.5 (variant) 1–4-4 affected some groups, the presence of other lineages could have influenced the detection patterns of *L. intracellularis* and PCV2.

Regarding the number of PRRSV genomic copies, 7 out of 9 groups classified as PRRSV quantile 4 belonged to either quantile 3 or 4 of *L. intracellularis* and PCV2. This indicates they had high quantities of *L. intracellularis* or PCV2 detected, while PRRSV was also detected in high quantities. This finding suggests that the presence of PRRSV in high amounts in a pig group might affect the dynamics of *L. intracellularis* or PCV2 detection, and vice-versa. Also, the fact that Low-performance groups started to significantly increase *L. intracellularis* positivity after a sequence of increased positivity of PRRSV detection from weeks 15 until 21 supports the association between these two pathogens. For PCV2, the synergetic effect of PRRSV enhancing replications for both viruses is well described in controlled trials (38, 48), supporting the higher quantiles of PCV2 in groups more affected by PRRSV in the field conditions of this study. However, from a bacteria standpoint, PRRSV has been correlated with the proliferation of pathogens belonging to the porcine respiratory diseases complex, such as *Streptococcus suis* (49), *Glaesserella parasuis* (50), and *Actinobacillus pleuropneumonia* (51). On the other hand, enteric bacteria are not commonly associated with PRRSV, and this study demonstrated higher detection of *L. intracellularis* associated with PCV2 and PRRSV in Low-performance groups. It is also important to highlight that clinical disease or mortality due to *L. intracellularis* was not observed by attending veterinarians, demonstrating the potential impacts of subclinical infection on performance and interaction with other pathogens (52). Also, there is evidence of a possible two-way communication between the gut and the airways, showing that respiratory issues can affect gut microbiome activity and that microbial compounds and metabolic products from the gut microbiome can influence respiratory immunity (53).

Even though PCR-positive fecal samples for *L. intracellularis* with Ct values below 20 have been associated with proliferative enteropathy lesions (54), there is a lack of knowledge on the association between oral fluids results and individual animals’ clinical outcomes and fecal shedding. Although oral fluids samples may contain environmental targets from within the collected pen (55), including feces, the association between individual pig fecal shedding and oral fluids results cannot be concluded from this study.

In addition, it is important to consider that all groups evaluated in this study received vaccines for PCV2 and *L. intracellularis*. As no groups remained unvaccinated, conclusions on vaccine efficacy cannot be made. As the efficacy of PCV2 and *L. intracellularis* vaccines has been established (20, 56) it is most likely that the severity of PCV2 and *L. intracellularis* infection would have been worse without vaccination. The higher pathogen detection of PCV2 and *L. intracellularis* was likely due to a higher infection pressure of these pathogens in the evaluated groups. Also, vaccination for PRRSV was not administered in any of the groups, which could have mitigated the impacts of PRRSV introduction on performance metrics and the consequences of exacerbation of other pathogens (32).Still, regarding the *L. intracellularis* vaccination, there is a possibility that the increased oral fluid positivity rate on sampling points 9–13 may be due to the vaccination since animals were vaccinated between weeks 5–11 of age and may be shedding vaccine through feces few days after vaccination (9).

It is essential to note the study’s limitation of having data from a single production system and a small sample size, as some factors might vary among different systems. In addition, considering this study was a prospective field trial performed in a large production system, variables such as biosecurity practices, environmental conditions, co-circulation of other pathogens or nutritional management practices could not be controlled, potentially confounding the observed performance. However, the findings of this study reinforce the importance of investigating the impact of multiple pathogens in farms, targeting a holistic approach by veterinarians to identify possible causes affecting pig flow, mainly when multiple pathogens are involved. Also, there was a limitation regarding the data after week 27 of age. In specific PRRSV 1st detection scenarios, there were no groups after this specific age for circumstances of the animal marketing strategy of the company. Therefore, comparing these sampling points for high and low performance groups must be interpreted cautiously. The study expands our understanding of PRRSV, PCV2 and *L. intracellularis* co-detections and the variables contributing to its impact on swine populations. As veterinarians, we often do not identify the reasons for variability among key performance indicators and variations in response to PRRS, which could be explained by co-infections playing a role in the disease dynamic. In the conditions of this study, groups affected by PRRSV and with higher detection of PCV2 and *L. intracellularis* had higher mortality and lower average daily gain performance. The study sheds light on the importance of evaluating the impact of co-detection in swine farms, even among organisms affecting different physiological systems, as in this study, enteric and systemic pathogens could be seen interacting, affecting key performance indicators.

## Data Availability

The original contributions presented in the study are included in the article/supplementary material, further inquiries can be directed to the corresponding author.
